# Comparing medical, dental, and nursing students’ preparedness to address lesbian, gay, bisexual, transgender, and queer health

**DOI:** 10.1371/journal.pone.0204104

**Published:** 2018-09-20

**Authors:** Madelyne Z. Greene, Katherine France, Edward F. Kreider, Emily Wolfe-Roubatis, Kevin D. Chen, Andy Wu, Baligh R. Yehia

**Affiliations:** 1 Department of OBGYN, University of Wisconsin-Madison School of Medicine and Public Health; Madison, WI, United States of America; 2 Department of Oral Medicine, University of Pennsylvania School of Dental Medicine, Philadelphia, PA, United States of America; 3 Department of Internal Medicine, Hospital of the University of Pennsylvania, Philadelphia, PA, United States of America; 4 School of Nursing, University of Pennsylvania, Philadelphia, PA, United States of America; 5 The Wharton School, University of Pennsylvania, Philadelphia, PA, United States of America; 6 Department of Business Administration, Harvard Business School, Boston, MA, United States of America; 7 School of Medicine, Johns Hopkins University, Baltimore, MD, United States of America; Pontificia Universidade Catolica do Rio Grande do Sul, BRAZIL

## Abstract

**Background:**

Lesbian, gay, bisexual, transgender, and queer (LGBTQ) populations face multiple health disparities including barriers to healthcare. Few studies have examined healthcare trainees’ perceptions of their preparedness to care for LGBTQ populations and none have compared perceptions of training across medicine, dental medicine, and nursing. We aimed to understand variations across disciplines in LGBTQ health by assessing medical, dental, and nursing students’ perceptions of preparedness across three domains: comfort levels, attitudes, and formal training.

**Methods:**

We developed a 12-item survey with an interprofessional panel of LGBTQ students from the schools of medicine, dental medicine, and nursing at a top-tier private university in the United States. Any student enrolled full time in any of the three schools were eligible to respond. We performed descriptive statistical analyses and examined patterns in responses using Kruskal-Wallis tests and an ordered logistic regression model.

**Results:**

1,010 students from the Schools of Medicine, Dental Medicine, and Nursing responded to the survey for an overall response rate of 43%. While 70–74% of all student respondents felt comfortable treating LGBTQ patients, fewer than 50% agreed that their formal training had prepared them to do so. Overall, 71–81% of students reported interest in receiving formal LGBTQ health education, though dental students were significantly less likely than medical students to report this interest (*OR* 0.53, *p*<0.01). Respondents who identified as LGBQ were significantly less likely than heterosexual students to agree that training was effective (*OR* 0.55, *p*<0.01) and that their instructors were competent in LGBTQ health (*OR* 0.56, *p*<0.01).

**Conclusion:**

Despite high comfort levels and positive attitudes towards LGBTQ health, most student respondents did not report adequate formal preparation. There were some significant differences between disciplines, but significant gaps in training exist across disciplines. Health professional schools should develop formal content on LGBTQ health and utilize this content as an opportunity for interprofessional training.

## Introduction

Lesbian, gay, bisexual, transgender, and queer (LGBTQ) individuals experience challenges in accessing care and achieving positive health outcomes.[1, 2] Compared to their heterosexual and cisgender peers, LGBTQ people are more likely to be uninsured, to delay or avoid medical care due to stigma, and to report poor overall health.[2, 3] LGBTQ patients cite discrimination in healthcare and providers’ lack of awareness of relevant health issues as reasons to delay or avoid care.[4] The largest survey of transgender individuals in the United States (U.S.) found that 28% of transgender respondents postponed medical care due to discrimination and 19% had been refused healthcare due to their transgender or gender non-conforming identity.[5] Transgender and gender nonconforming individuals are less likely to have health insurance or regular primary care providers than their cisgender peers.[6] These disparities are, at least in part, due to bias and lack of training among healthcare providers, including physicians, dentists, and nurses. Some recent studies suggest that access to high quality, inclusive healthcare is increasing,[7] but further improvements in the training of all health professionals will be necessary to improve health outcomes among LGBTQ populations.

Recognizing these disparities, the U.S. Department of Health and Human Services’ Healthy People 2020 program set goals to improve the health, safety, and well-being of LGBTQ individuals.[7] In 2011, the National Institute of Health (NIH) also commissioned the National Academy of Medicine (NAM) to conduct a comprehensive review of the health needs of LGBTQ populations.[2] The report highlighted the paucity of and necessity for research focused on LGBTQ patients and communities. More recently, the National Institute on Minority Health and Health Disparities (NIMHD) announced the inclusion of sexual and gender minorities as a “health disparity population” for health research.[8]

Several studies have evaluated LGBTQ health training and education in medical schools. Both the American Medical Association (AMA)[9] and Association of American Medical Colleges (AAMC)[10] specifically recommend the inclusion of LGBTQ-focused topics in medical education to adequately prepare clinicians. The AAMC has also developed a curriculum for “Implementing Curricular and Institutional Climate Changes to Improve Healthcare for Individuals Who are LGBT, Gender Nonconforming, or Born with DSD,” which outlines the health needs of LGBTQ individuals and strategies for medical schools in designing curricula around LGBTQ health.[10] However, a 2011 survey of 176 U.S. medical school deans reported a median of two hours of LGBTQ-related content, with 44.1% of deans reporting “poor” or “very poor” coverage of LGBTQ-specific topics.[11] In an online survey of 4,262 medical students in the U.S. and Canada, most respondents (67.3%) evaluated their LGBTQ-related curriculum as “fair” or worse. After receiving specific education on LGBTQ health, 62.6% of these students felt more prepared and 46.3% felt more comfortable caring for LGBTQ patients.[12] Some limited evidence suggests that inclusion of LGBTQ content can effectively improve patient care. For example, a survey of one urban U.S. medical school found that students who had more clinical encounters with LGBTQ patients were significantly more likely to take comprehensive health histories, hold positive attitudes toward LGBTQ patients, and possess knowledge of LGBTQ health than students with little or no clinical exposure.[13]

Recent studies have also revealed underlying bias and discrimination against LGBTQ populations in medical educational settings. One 2017 study found that 41.7% of medical students had witnessed discriminatory behaviors toward LGBTQ students by their peers and superiors.[14] In another large study of medical students, 45.8% of heterosexual first-year students exhibited explicit bias and 81.5% demonstrated implicit bias toward LGBTQ individuals.[15] A third study tested relationships between medical students’ implicit sexual orientation bias and various aspects of their medical school environment. They concluded that medical schools may reduce bias among students by reducing negative role modeling among faculty, improving the overall climate of diversity, and improving formal student preparedness in LGBTQ health-related topics.[16]

Fewer studies have evaluated LGBTQ-related content in U.S. dental schools, or attitudes among dental students. However, the existing evidence suggests that dental medical training may include significantly less LGBTQ-related content. To provide high quality oral healthcare, dental professionals must be prepared to address issues of access to care and inclusion, usage of inclusive language including the preferred name and pronouns of transgender patients, and recognizing the family structures of LGBTQ individuals. Additionally, dental professionals must elicit appropriate information and make clinical decisions related to pharmacologic agents that LGBTQ clients may use (e.g. gender-affirming hormone therapy). This includes awareness of the side effect profile associated with such agents including possible oral effects such as increased inflammatory reactions, risk for autoimmune reactions in the oral cavity, and medication-related xerostomia.[17] Despite the need for LGBTQ competency in dental medicine, in a 2004 survey of 47 U.S. dental schools, 49% of administrators reported that their curriculum contained between zero and two hours of LGBTQ-related content.[18] A 2009 survey of student leaders from 30 dental schools in the U.S. and Canada found that only 13.3% agreed that their education prepared them well to treat LGBTQ patients.[19] Among U.S. and Canadian dental school administrators, 62.9% reported that their school had a written policy to protect LGBTQ students, and 83.3% reported a general anti-discrimination policy. However, 72.2% disagreed or strongly disagreed that it was important to provide specialized academic support for LGBTQ students.[20]

Research on LGBTQ-related curriculum in nursing schools is also limited. In one study, scores on a 15-item LGBT health questionnaire improved from 13.48 to 14.67 after a brief learning module.[21] Another study surveyed 1,231 nursing school faculty members about LGBTQ curricular content and their preparedness to teach it.[22] While 70% of faculty respondents indicated they were moderately or fully ready to teach LGBTQ topics, only 29% reported they had full or adequate knowledge of LGBTQ health issues.

To our knowledge there have been no published studies comparing LGBTQ curriculum and attitudes across medical, dental, and nursing schools. However, increasing attention on interdisciplinary education highlights the value of comparing how these three disciplines address preparedness to care for LGBTQ patients. By comparing three individual schools at one large institution, we can gain perspective on the strengths and weaknesses of each and identify the ways health professional schools may work together to improve efficiently. Comparing the attitudes and experiences of students at different health profession schools may also suggest some underlying differences in the current attitudes of providers in each field.

The purpose of this study was to assess medical, dental, and nursing students’ perceptions of their preparedness to care for LGBTQ patients by measuring their comfort levels with, attitudes toward, and formal training in LGBTQ health. We hypothesized that responses in all three domains would vary by both discipline and demographic groups. Specifically we hypothesized that students identifying as LGBTQ would be more comfortable treating LGBTQ patients and have more positive attitudes towards LGBTQ populations, but would be less likely to agree that their formal training in LGBTQ health was adequate.

## Methods

We conducted a cross-sectional survey of students in the Schools of Medicine, Dental Medicine, and Nursing at the University of Pennsylvania between August and November 2014. Students enrolled in any degree program at these schools were eligible for the study.

### Recruitment and procedures

Respondents were recruited through emails from the study team and school administrators and through distribution of surveys during large course meetings. Students had multiple opportunities to complete the survey either on paper or online but were instructed to do so only once. The study’s aims and a statement about voluntary participation were shared verbally, in all emails, and in writing on the survey tool.

### Ethics statement

The authors disclose no conflicts of interests.

The University of Pennsylvania IRB approved this investigation (Protocol #820037) as an exempt study because the study did not require the collection of any identifiable data from any participants. This designation waived the IRB need for formal written consent. Respondents read a statement which informed them that the study was completely voluntary, summarized the purpose of the study, and how their data would be used. Respondents were informed that by completing the survey they were offering their consent to participate. Students were encouraged to contact the study team with questions or concerns. We did not collect any identifying information in order to ensure anonymity and reduce refusal rates due to related concerns. All survey data were stored in the REDCap online data application.[23] The IRB approved this consent procedure as submitted in our IRB protocol.

### Survey tool

We developed a 12-item survey to assess students’ perspectives on how both the formal and informal curricula at their respective schools prepared them to care for LGBTQ patients. The “formal curriculum” refers to planned programs and learning experiences and the knowledge and skills that students are explicitly expected to learn. The “informal curriculum” refers to ideas and lessons conveyed through policies, role-modeling, and institutional culture.[24] Our 12-item survey tool evaluated self-perception of preparedness across three domains that frame our broad understanding of preparedness to care for LGBTQ populations: 1) comfort with providing care to LGBTQ populations, 2) attitudes towards LGBTQ populations and health, and 3) formal training in LGBTQ healthcare. These domains reflect the recommendations of AAMC leaders in a recent publication that institutions make active changes to both the formal and informal aspects of healthcare training curricula in order to effectively enhance provider competency and improve patient care.[25] A recent review of educational interventions in LGBTQ health also supports that both knowledge and attitudes towards LGBTQ populations and health are important indicators of provider preparedness.[26] This review and other observational studies confirm that comfort, attitudes, and formal knowledge are all likely to play important roles in improving patient care.[26–28] Table 1 displays the survey items in their respective domains.

**Table 1:** Survey items given to all respondents.

Survey Items
Comfort Level
1. I feel comfortable discussing sexual health with my patients.
2. I feel comfortable treating lesbian, gay, bisexual, and queer patients.
3. I feel comfortable treating trans-identified patients.
Attitudes
4. I believe it is the responsibility of all healthcare providers to care for LGBTQ patients.
5. I can tell if my patient is lesbian, gay, bisexual, or queer by looking at them.
6. I can tell if my patient is trans or gender-non-conforming by looking at them.
7. It is more challenging to discuss sexual health with LGBTQ patients than with heterosexual or non-transgender (cis-gender) patients.
Formal Training
8. My training at Penn has prepared me to care for LGBTQ patients.
9. If I have a question regarding LGBTQ care, I know where to look for the answer.
10. My school/program has incorporated LGBTQ related content into a variety of courses.
11. My instructors demonstrate competency in caring for LGBTQ patients.
12. I am interested in receiving further education at Penn about LGBTQ health issues.

We developed individual survey items de novo and reviewed them for face and construct validity with an interprofessional group of LGBTQ and allied students. Questions were explicitly formulated to be applicable across healthcare disciplines based on the input of our interprofessional group. We also distinguished between sexual and gender minorities in all survey items since all three domains are likely to vary based on the distinction of sexual orientation and gender identity. Respondents indicated their level of agreement with each survey item on a 5-point scale from “strongly agree,” “to “strongly disagree.” We also collected the healthcare discipline, age, gender identity, sexual orientation, and race/ethnicity data.

### Data management and statistical analysis

We first conducted descriptive analyses of survey data. Since the meaningful distinctions between response options “strongly agree” and “agree were unclear, we combined responses into three groups: “agree/strongly agree,” “undecided,” and “disagree/strongly disagree.” All statistical analyses are based on these combined response groups. Since the survey tool had not been formally validated we analyze each survey item individually throughout the analysis.

Several demographic variables were grouped for analysis. We divided age data into four categories; ≤22 years old, 23–25 years old, 26–29 years old, and ≥30 years old. Respondents could indicate multiple responses for race/ethnicity, sexual orientation, and gender identity, but we created mutually exclusive categories for analysis. Respondents were categorized as an underrepresented minority (URM) if they indicated Black, Latino/Hispanic, or Native American/Alaskan Native/Pacific Islander, to reflect common definitions of underrepresentation in healthcare fields. All respondents with any non-heterosexual sexual orientation (which included lesbian, gay, bisexual, and queer identities) were grouped as “LGBQ” for analysis to achieve enough statistical power for meaningful findings. We distinguish “LGBQ” respondents from gender minority respondents because gender identity is distinct from sexual orientation and transgender individuals also have a distinct sexual orientation. Four respondents marked “other” as their gender identity. Two of these individuals also marked male or female gender and were grouped accordingly, while the two respondents who chose only “other” were grouped in an “other” gender category.

We then compared responses to each question across schools using both the non-parametric Kruskal-Wallis test with α set to 0.05 and an ordered logistic regression model. The model estimates the effects of demographic variables, including school, on the likelihood that respondents agreed or strongly agreed with each survey item. Regression coefficients are reported as odds ratios (OR). Statistical analysis was performed using Stata (StataCorp, Stata Statistical Software: Release 14, College Station, TX) and R analysis software (The R Project for Statistical Computing, 3.0, Vienna, Austria).

## Results

A total of 1,010 students completed the survey. Table 2 describes characteristics of the sample. The overall survey response rate was 43%, with a response rate of 76% in the School of Medicine (*n* = 495), 24% in the School of Dental Medicine (*n* = 127), and 33% in the School of Nursing (*n* = 388). The variation in response rates is likely a product of the convenience sampling method used for data collection and may reflect students in each school were given opportunities to complete the survey. Medical students, for example, were most commonly recruited during required lecture courses that are typically taken by entire cohorts of students in each year. Dental students were reached more commonly through email since the study team was not offered the opportunity to recruit during courses. The mean age for all respondents was 24.7 years and 18% of respondents identified as underrepresented minorities. Across schools, 65% percent of respondents (*n* = 657) identified as female, and 13% (*n* = 134) identified as lesbian, gay, bisexual, or queer, although distributions varied between schools. The School of Nursing had a higher percentage of female respondents (88%, *n* = 341) compared to the School of Medicine (62%, *n* = 237) and School of Dental Medicine (48%, *n* = 79). A smaller proportion of Medical students identified as LGBTQ (9%, *n* = 47) compared to dental (17%, *n* = 22) and nursing (17%, *n* = 65) student respondents.

**Table 2:** Demographic characteristics of the sample (N = 1,010).

Demographic variable	Number of respondents (N)	Percent (%)
Total respondents	1,010	100
Age		
≤22	252	25.8
23–25	409	41.9
26–29	225	23.1
≥30	89	9.1
Race/Ethnicity		
White	587	58.3
Asian	235	23.3
Black	69	6.9
Latino	105	10.4
Native	11	1.1
Gender		
Male	349	34.6
Female	657	65.2
Other	2	0.20
Sexual Orientation		
Heterosexual	871	86.7
LGBQ	134	13.3
School		
School of Medicine	495	49.0
School of Dental Medicine	127	12.6
School of Nursing	388	38.4

### Overall responses

**Fig 1:**
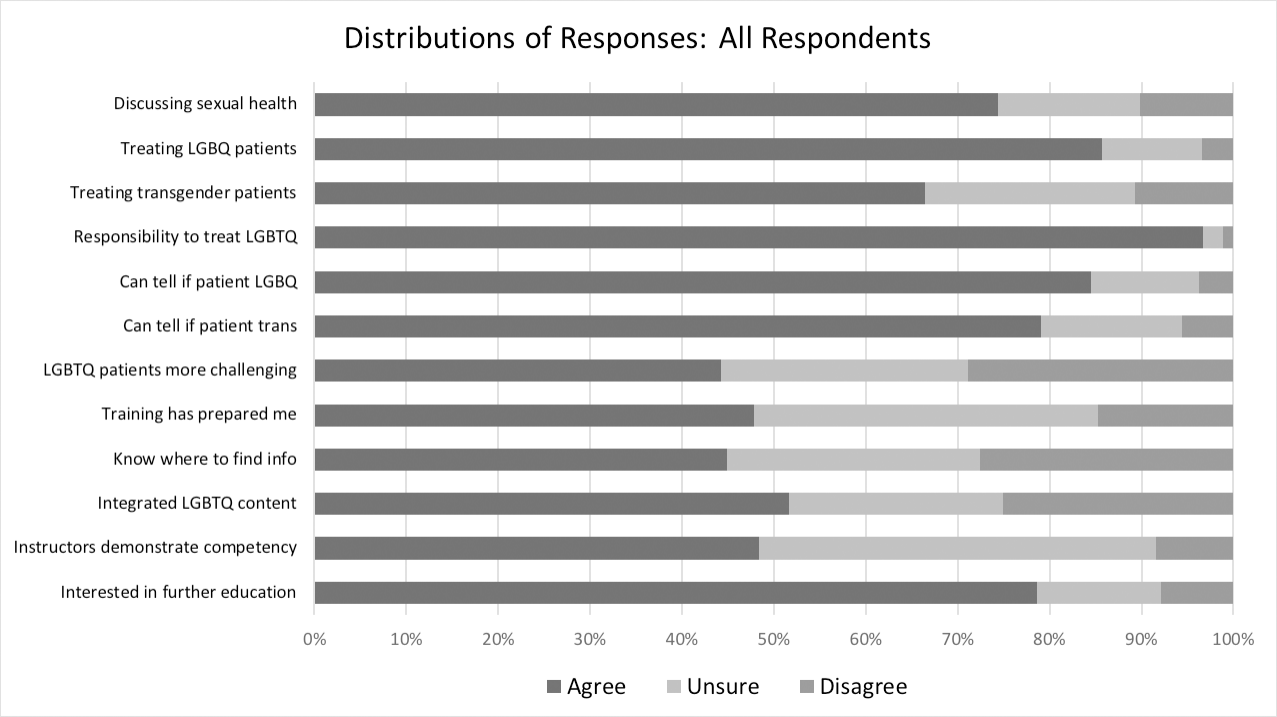
Distribution of survey responses by school. The directionality of responses to items 5, 6, and 7 were reversed in this figure so that A/SA responses consistently indicate higher comfort levels, positive attitudes, or good formal training in LGBTQ health. This figure uses abbreviated forms of survey items: see Table 2 for full survey items.

Fig 1 displays response distributions for each item from all respondents. Respondents reported generally high comfort levels with and positive attitudes toward LGBTQ health. Overall, 86% (*n* = 861) of respondents agreed that they felt comfortable treating lesbian, gay, bisexual, or queer (LGBQ) identified patients. Fewer respondents (66%) agreed that they felt comfortable treating trans-identified patients. An overwhelming majority of respondents (97%, *n* = 972) agreed that all healthcare providers have a responsibility to care for LGBTQ patients. Only 29% of respondents (*n* = 290) agreed that discussing sexual health with LGBTQ patients is more difficult than with heterosexual and cisgender respondents. Two items about identifying sexual minority and gender minority individuals by sight alone assessed the salience of stereotypes among respondents. Based on the framing of these items (items #5 and #6), agreement suggested a stereotypical attitude while disagreement suggested LGBTQ patient competency. Overall, 84.5% of respondents disagreed that they could identify LGBQ patients by sight and 79.0% disagreed that they could identify transgender patients by sight.

Fewer respondents agreed with survey items focused on formal training in LGBTQ health. Forty-eight percent of respondents (*n* = 481) agreed that their training had prepared them to care for LGBTQ patients, and 45% of respondents agreed that they knew where to look for information regarding LGBTQ health. Overall, 79% of respondents (*n* = 671) agreed that they were interested in receiving more education about LGBTQ health. Students’ perceptions of instructor competency in LGBTQ health was low, with only 48% of students reporting that their instructors had demonstrated LGBTQ health competency. Similarly, 52% of respondents reported that their school had integrated LGBTQ content in to the formal curriculum. However, most students (79%) were interested in further education on LGBTQ health topics in their professional program.

### Variation across participant disciplines

We performed Kruskal-Wallis tests to measure differences in the distribution of responses between schools, and found significantly different response distributions (*p*<0.05) for all survey items except items 8, 9, and 12 (all in the formal training domain). Most of these differences reflect differences between dental students and other respondents. For example, significantly fewer dental students (52%, *n* = 63) agreed that they felt comfortable discussing sexual health than medical students (79%, *n* = 388) and nursing students (76%, *n* = 295). Significantly fewer dental students (25%, *n* = 32) agreed that their instructors demonstrated competence compared to medical (46%, *n* = 227) and nursing students (59%, *n* = 227). Additionally, only 13% (*n* = 16) of dental students agreed that their curriculum had incorporated LGBTQ related content compared to 58% (*n* = 287) and 55% (*n* = 215) of medical and nursing students, respectively. While only 3% of medical and nursing students (*n* = 17; *n* = 13) agreed that they could visually identify whether a patient was LGBQ, 6% of dental students (*n* = 8) agreed. Similarly, while only 4% (*n* = 24) of medical and 5% (*n* = 17) of nursing students agreed that they could tell whether a patient was transgender or gender nonconforming by sight, 12% (*n* = 15) of dental students agreed. Conversely, more dental students (92%, *n* = 118) agreed that they were comfortable treating LGBQ and trans patients than medical (83%, *n* = 411) and nursing students (86%, *n* = 334).

Table 3 shows the results of the ordered logistic regression model, which was used to estimate the effects of demographic variables and specific discipline on the likelihood of agreeing with each survey item individually.

**Table 3:** Results of the ordered logistic regression model. Reported values are adjusted Odds Ratios (OR) of agreeing with each of the twelve included survey items. Respondents who indicated gender identifications other than female or male were excluded from this analysis, though their presence at the studied institution should be noted.

	Comfort Level			Attitudes				Formal Training				
Variable	1. Discus-sing sexual health.	2. Treating LGBQ patients.	3. Treating trans patients.	4. Responsib-ility to treat LGBTQ patients.	5. Can tell if a patient is LGBQ.	6. Can tell if a patient is trans-gender.	7. LGBTQ patients more challen-ging.	8. Prepared to treat LGBTQ patients.	9. Know where to find LGBTQ info.	10. School has inte-grated LGBTQ content.	11. Instructors demon-strate competen-cy.	12. Interested in further LGBTQ education
*n*	*967*	*965*	*826*	*966*	*966*	*967*	*967*	*966*	*966*	*965*	*966*	*827*
Age	1.058***	1.026	1.034*	1.027	0.97	0.982	0.944***	0.973	0.996	0.961**	0.944***	1.011
(continuous)												
Gender												
Male (ref)	1	1	1	1	1	1	1	1	1	1	1	1
Female	0.871	0.669**	0.807	1.508*	0.574***	0.614***	0.78	0.861	0.732*	0.886	0.756	2.175***
Sexual Orientation												
Heterosexual (ref)	1	1	1	1	1	1	1	1	1	1	1	1
LGBQ	1.407	2.203***	2.039***	3.974***	0.659*	0.637*	0.373***	0.554**	1.1	0.581**	0.555**	5.789***
Race/Ethnicity												
White (ref)	1	1	1	1	1	1	1	1	1	1	1	1
Asian	0.665**	0.508***	0.681*	0.658*	1.765***	1.679**	1.332*	0.549***	0.823	0.688*	0.660**	1.069
Black	1.532	0.917	0.937	0.955	1.323	1.173	0.823	0.657	0.956	0.619	0.77	1.41
Latinx/Hispanic	1.008	1.121	1.107	0.797	0.793	0.771	0.822	0.853	0.939	0.871	0.919	1.349
School												
Medicine (ref)	1	1	1	1	1	1	1	1	1	1	1	1
Dental	0.273***	1.878**	2.339***	0.794	2.448***	2.052***	0.534***	0.877	1.134	0.114***	0.385***	0.534***
Nursing	1.024	1.077	1.664***	0.414***	1.014	0.993	0.641**	0.947	1.024	0.923	1.536**	0.735

* = *p*<0.05.

** = *p*<0.01.

*** = *p*<0.001.

This model confirms our findings from Kruskal-Wallis tests that dental students were less likely to be comfortable discussing sexual health (*OR* 0.27, *p*<0.001) than medical students, but more likely to be comfortable treating LGBQ (*OR* 1.88, *p*<0.01) and transgender patients (*OR* 2.34, *p*<0.001). Both dental (*OR* 0.534, *p*<0.001) and nursing (*OR* 0.641, *p*<0.01) student respondents were more likely than medical students to indicate that discussing sexual health with LGBTQ patients was more difficult than with heterosexual patients. Dental students were also again less likely to agree that LGBTQ content was integrated into their program (*OR* 0.11, *p*<0.001) and that their instructors demonstrated competency in this area (*OR* 0.39, *p*<0.001). Nursing students were more likely to agree that their instructors demonstrated competency in LGBTQ health (*OR* 1.54, *p*<0.01) than medical students. Dental students were less likely to report interest in further training than medical students (*OR* 0.53, *p*<0.001).

### Variation across demographic characteristics

Results from the logistic regression model also suggest that demographic factors such as participant gender, sexual orientation, and race/ethnicity may be associated with the likelihood of agreeing with the survey items. The two respondents remaining in an “other” gender identity group were excluded from the regression analysis. Therefore, comparisons by participant gender include only male and female respondents. Compared to male respondents, female (*OR* 1.51, *p*<0.05) respondents were more likely to agree that all healthcare providers have a responsibility to care for LGBTQ patients. Female respondents were also more likely to disagree that one can identify a sexual or gender minority individual by sight alone (*OR* 0.57, *p*<0.001; *OR* 0.61, *p*<0.001). Finally, female respondents were also twice as likely as male respondents (*OR* 2.18, *p*<0.001) to express interest in further education on LGBTQ health topics.

The odds of agreeing with several survey items were also impacted by participant sexual orientation. Respondents with any LGBQ identity were overall more likely to endorse comfort with and positive attitudes towards LGBTQ populations. LGBQ respondents were more than twice as likely to agree that they were comfortable caring for LGBQ (*OR* 2.20, *p*<0.001) and transgender (*OR* 2.04, *p*<0.001) patients than were heterosexual respondents. LGBQ (*OR* 3.97, *p*<0.001) respondents were also more likely to agree that all healthcare providers have a responsibility to care for LGBTQ patients. Similarly, LGBQ respondents were less likely to agree that they could recognize sexual and gender minorities (*OR* 0.66, *p*<0.05; *OR* 0.64, *p*<0.05) by sight alone. LGBQ respondents were less likely to agree that discussing sexual health with LGBTQ patients is more difficult (*OR* 0.373, *p*<0.001). However, LGBQ respondents generally had more negative perceptions of their formal training in LGBTQ health. LGBQ respondents were less likely to agree that their programs had prepared them to care for LGBTQ patients (*OR* 0.55, *p*<0.01), that LGBTQ content was integrated into their programs (*OR* 0.58, *p*<0.01), and that their instructors demonstrated competency in LGBTQ health (*OR* 0.56, *p*<0.05). LGBQ respondents were over 5 times as likely (*OR* 5.79, *p*<0.001) to report interest in receiving additional LGBTQ-specific education.

In addition, participant race/ethnicity was found to be associated with the odds of agreeing with some survey items. Respondents in the Asian race/ethnicity group were less likely to agree with all three survey items reflecting comfort levels compared to white respondents. This group of respondents were more likely to agree that they could identify gender and sexual minority patients by sight alone (*OR* 1.77, *p*<0.001; *OR* 1.68, *p*<0.01) and that discussing sexual health with LGBTQ patients was more difficult (*OR* 1.33, *p*<0.05). We found no statistically significant differences in odds of agreeing with survey items between white and URM respondents.

## Discussion

To our knowledge this was the first study to examine and compare medical, dental, and nursing students’ perceptions of their preparedness for caring for LGBTQ populations. Our results demonstrate that health professional students generally hold positive attitudes towards caring for LGBTQ populations and have interest in receiving more LGBTQ-focused training. However, respondents reported mixed evaluations of the formal preparation they had received.

### Variation across disciplines

Dental students specifically displayed slightly less positive and more stereotypical attitudes towards LGBTQ populations and had less positive perceptions of their formal training in LGBTQ health. There are several possible explanations for these findings. There may in fact be a more significant gap in LGBTQ health content and instructor competency in this School of Dental Medicine compared to the School of Medicine and School of Nursing. There was also less interest in LGBTQ-specific training among dental students. However, dental student respondents had the highest odds of agreeing that they were comfortable treating LGBQ and transgender patients. Alternatively, the high reported levels of comfort treating LGBTQ patients in this survey may be attributable to selection bias, in which dental students who already felt more comfortable with LGBTQ populations and therefore perceived less need for additional training in LGBTQ health were more likely to complete the survey. The significantly lower response rate in the School of Dental Medicine also suggests that selection bias may disproportionately affect findings about dental student respondents.

These seemingly contradictory findings may also reflect the misconception that LGBTQ-specific training is not relevant to or required for high-quality dental practice. In fact, the inclusion of LGBTQ healthcare topics in dental curricula is important for various clinical skills. First, dental practitioners, like all healthcare professionals, need to be aware of and be able to address barriers to healthcare that are commonly experienced by marginalized populations. Dental practitioners must also understand the effects of relevant medical history or medication use on dental care, and effectively screen for oral infections and diseases for which parts of the LGBTQ population may be at higher risk. These include oral lesions that result from sexually transmitted infections as well as oral complications of other health disparities faced by the LGBTQ population, such as decreased healthcare usage, increased substance abuse, and others. It is important that a dentist can effectively elicit relevant information from all patients by asking appropriate questions and speaking knowledgeably about sexual health with all patients.

The Commission on Dental Accreditation’s accreditation standards include standards for cultural competence.[29] The American Dental Education Association’s Diversity and Inclusion Advisory Committee also recommends that dental education programs include cultural competency education and “a broader diversity agenda that goes beyond race/ethnicity.”[30] While these standards and recommendations can be interpreted to include gender and sexuality topics, they do not specifically mention or require coverage of LGBTQ populations. National dental education organizations should identify and name relevant LGBTQ health topics and schools of dental medicine should outline specific curricular goals related to LGBTQ health. These actions by national organizations can help lead to more effective and complete care of LGBTQ patients in dental care settings.

Differences in trainees’ perceptions of their preparedness to care for LGBTQ populations across disciplines in this study highlight the potential value of interprofessional learning and resource-sharing when addressing LGBTQ-focused content. The National Academy of Medicine has identified interprofessional learning as a key strategy for effective health professional education.[31] Improving LGBTQ health outcomes and narrowing disparities will require interprofessional and healthcare team-based interventions. Using interprofessional approaches to improving LGBTQ health education will allow individual schools to benefit from others’ resources and successes and increase opportunities for exposure to LGBTQ populations during clinical training. For example, schools could develop cross-listed courses focused on LGBTQ health topics that are available to students across health professions. Students could engage in interprofessional clinical and simulation-based training experiences focused on LGBTQ health that teach both clinically relevant information but also communication and teamwork skills.[32] Interprofessional programs also have the potential to increase the visibility of LGBTQ health initiatives within a university or health system, further improving aspects of the training environment that may impact students’ comfort level with and attitudes towards LGBTQ populations.

### Variation across demographic factors

Respondents’ gender and sexual orientation also impacted responses in this study. For example, holding other factors constant, female trainees were more likely to agree that all healthcare providers have a responsibility to treat LGBTQ patients, and be interested in further LGBTQ-focused education. Our data cannot explain what is driving such differences, but it is possible that female-identified trainees have more personal experience with the impact of gender on health and therefore recognize the importance of LGBTQ-specific health education. Further research on the impact of provider gender identity on patient care among LGBTQ individuals is warranted.

LGBQ respondents also reported poorer perceptions of their formal training in LGBTQ health than those who identified as heterosexual. This population may have higher expectations for such training, or may be more attentive to the quality of LGBTQ content in their training. LGBQ respondents also reported more comfort with treating LGBQ and transgender patients, possibly due to personal or extracurricular clinical experiences in settings with LGBTQ patient populations. Health professional schools may be able to draw on the experience and expertise of LGBTQ and allied students to develop formal content, clinical immersion opportunities, and a safe and welcoming climate. Ongoing collaboration with these student leaders can help identify deficiencies in the formal curriculum and foster innovative solutions and initiatives to include LGBTQ health. Once identified, however, it is crucial that the work of students be fully institutionalized via course content, special educational opportunities, programmatic policies, and elsewhere, so that these improvements become effective long-term solutions. Changing institutional culture to fully include LGTBQ health content requires that *all* students be consistently exposed to LGBTQ content and that schools maximize opportunities for clinical encounters with LGBTQ patients. This requires the engagement and commitment of both faculty and school administrators, and permanent integration of LGBTQ-related topics into health professional training.

Some survey items were also significantly impacted by the race/ethnicity of respondents. Holding other variables constant, respondents in our study who identified their race/ethnicity as Asian were significantly less likely to be comfortable with LGBQ and transgender patients, agree that all healthcare providers were responsible for caring for LGBTQ patients, and had poorer perceptions of their formal training in LGBTQ health compared to their white peers. Again, our data cannot explain the cause of these differences, but differences in comfort levels may reflect different social norms around sexuality and LGBTQ populations. Poorer perceptions of their formal training in LGBTQ health may represent a similar attentiveness to content related to marginalized populations among nonwhite trainees. While no other race/ethnicity identities were statistically significantly associated with the odds of agreeing with survey items, all nonwhite groups had point estimates less than one for all survey items that evaluated formal training (see Table 3). Some research has suggested that a more diverse healthcare workforce may have the potential to mitigate some healthcare disparities, especially those related to access.[33, 34]

### Study limitations

Our study was limited by several factors. For instance, we achieved a relatively low response rate (24%) from the School of Dental Medicine and convenience-sampling techniques limit the representativeness of the sample. In addition, we conducted this study at a single large, private university in the northeastern U.S., which may attract a non-representative student body with particular pre-existing attitudes towards LGBTQ populations. Investigators should extend this research to include representative samples of medical, dental, and nursing trainees throughout the U.S. Additionally, we did not formally validate our survey prior to data collection, though items were reviewed by an interprofessional group of LGBTQ and allied students. Larger studies would benefit from a validated instrument to measure aspects of curriculum, including formal evaluation of unconscious and conscious bias against LGBTQ populations among health professional students.

It is also likely that social desirability bias had some effect on our findings. That is, respondents may not have responded honestly about negative attitudes towards or discomfort with LGBTQ individuals because they felt pressured to express acceptance of these populations due to social norms. Healthcare providers may be specifically subject to this bias. Students with positive attitudes toward LGBTQ populations or more knowledge of LGBTQ-related healthcare topics may have been more likely to respond to the survey. We made efforts to ensure anonymity of all respondents in order to mitigate the impact of this social desirability bias, but we recognize that it may still be present. By offering multiple opportunities to complete the survey to maximize response rate, this survey also was susceptible to redundant sampling. With de-identified response data, we were not able to delete any duplicate respondents, but all potential respondents were consistently instructed only to complete the survey once, both verbally and in writing.

Our study was also limited by relatively small sample sizes of LGBTQ respondents. This required us to combine all sexual minority identities into the “LGBQ” group for analysis. Our sample did not include enough transgender respondents to provide a meaningful group for analysis. It may be true that respondents identifying as lesbian, gay, bisexual, queer, or other identities may have significantly different perceptions of their training in LGBTQ healthcare, but we believe that a combined sexual minority group (LGBQ) are likely to represent a meaningful analytic group and that these potential differences are more likely to be at the level of specific LGBTQ content, which our study did not aim to evaluate.

Our survey did not collect certain data that may also be important in understanding healthcare trainees’ perceptions of preparation in LGBTQ health. For instance, medical trainees with different intended areas of medical specialization may have different comfort levels with or attitudes towards LGBTQ patients. However, these differences could be pre-existing, as opposed to a reflection of their pre-professional training. Regardless of the cause of these potential differences, though, efforts to improve clinical care will need a basic understanding of the preparation of clinicians practicing in specific specialties. Additionally, we did not analyze our data by trainees’ level of education. Nursing students are typically at a different stage of post-secondary education than are medical and dental students. The inherent differences between medical, dental, and nursing schools, including required number of years of training, different degree programs, and undergraduate or graduate-level training, prevented this analysis from being meaningfully included in the current study. Our findings do, however, support further investigation of how informal and formal curriculum shape comfort, attitudes, and knowledge throughout the course of healthcare education.

### Recommendations

The results of this survey further knowledge of LGBTQ healthcare education by comparing preparedness across three health professional schools. The results suggest a need for continued improvements in curriculum and development of validated evaluation tools for health professional curricula. Findings from this study show that medical, dental, and nursing students report similar issues in both the informal and formal curricula related to LGBTQ populations. Our findings suggest that interprofessional efforts to improve LGBTQ health training are warranted. Interprofessional approaches may be specifically valuable in their capacity to capitalize on limited resources and effect long-term change in trainee’s behaviors. As mentioned above, several previous studies have evaluated the short-term impact of brief educational and integrated curricular interventions on LGBT health knowledge, but to our knowledge, no studies have included long-term follow up to track objective aspects of patient care among those exposed to these educational interventions.

As healthcare training institutions work to improve curricula, they should also consider the prevalence of LGBTQ patients and professionals in their own communities. Institutions should foster inclusive environments and respect the expertise of these individuals and communities.

Interventions can also be made in individual programs to improve LGBTQ healthcare training. LGBTQ-related content in all healthcare training programs should appear as distinct topics, such as hormonal and surgical gender affirmation, the increased risk of mood disorders in certain LGBTQ communities due to social isolation and discrimination, health disparities affecting LGBTQ populations including increased alcohol, drug, and tobacco use, and appropriate use of language and gender pronouns. This content should also emerge through increasing visibility of these populations within more general topics. For example, case studies introduced throughout the curriculum should include LGBTQ people and families, and courses that focus on patient-provider communication, building rapport, or professionalism should include specific issues faced by LGBTQ populations. This content should be evidence-based and should avoid further stigmatization of LGBTQ people while addressing difficult and sensitive topics through contextualization.

## Supporting information

S1 Table Complete participant data. This table includes responses to all survey items from all student participants.
(XLSX)

S1 Fig Survey items. This figure depicts the survey administered to respondents in the School of Dental Medicine; respondents in the School of Medicine and School of Nursing responded to the same survey items that referred to their respective schools.
(PDF)
