# Supplementary material for: Comparing medical, dental, and nursing students’ preparedness to address lesbian, gay, bisexual, transgender, and queer health
Source: PLoS One. 2018 Sep 20;13(9):e0204104. doi: 10.1371/journal.pone.0204104 (PMC6147466; doi:10.1371/journal.pone.0204104)
Supplement: S1 Fig — This figure depicts the survey administered to respondents in the School of Dental Medicine; respondents in the School of Medicine and School of Nursing responded to the same survey items that referred to their respective schools. (PDF) [file pone.0204104.s002.pdf]

School of Dental Medicine - LGBTQ Climate Survey

This survey is part of a research study being conducted at the University of Pennsylvania, and asks questions about your perceptions of and experiences with lesbian, gay, bisexual, trans, and queer (LGBTQ) individuals and education at Penn. The goal of this survey to is to evaluate the climate at Penn's health professional schools. Please only complete this survey once. Participation in this research is completely voluntary, and completion and submission of the survey implies your consent to participate.

The survey is anonymous and confidential; no identifiable information will be recorded and your answers will never be reported individually. You can reach the researchers at PennNursesPUSH@gmail.com for questions or concerns about your participation in the study, and information about on-campus Counseling and Psychiatric Services (CAPS) is available upon request.

The following survey asks questions about your perceptions of and experiences with lesbian, gay, bisexual, trans, and queer (LGBTQ) individuals and education at Penn. Your participation in this survey will help us better understand and improve LGBT health and visibility at Penn. Participation is voluntary and no identifying information will be collected.
Willingness to complete the survey indicates consent to participate in the research study.

If you consent to participate, click yes below.

- ◯ Yes
- ◯ No

Please indicate your year in the School of Dental Medicine (PDM)

- ◯ First Year
- ◯ Second Year
- ◯ Third Year
- ◯ Fourth Year

2 Please indicate your age in years: ________________________________

3 Please indicate your gender (select all that apply):

- ☐ Trans (male to female)
- ☐ Trans (female to male)
- ☐ Genderqueer
- ☐ Woman
- ☐ Man
- ☐ Other

If other, please explain: ________________________________

4 Please indicate your sexual orientation (select all that apply):

- ☐ Lesbian
- ☐ Gay
- ☐ Bisexual
- ☐ Queer
- ☐ Straight/heterosexual
- ☐ Asexual
- ☐ Other:

If other, please explain: ________________________________

5 Please indicate your race/ethnicity (select all that apply):

- ☐ Asian
- ☐ Black
- ☐ Latino/Hispanic
- ☐ Native American/Alaskan Native/Pacific Islander
- ☐ White
- ☐ Other:

If other, please explain: ________________________________

29 My training at Penn has prepared me to care for LGBTQ patients.

◯ Strongly Agree
◯ Agree
◯ Undecided
◯ Disagree
◯ Strongly Disagree

32 If I have a question regarding LGBTQ care, I know where to look for the answer.

◯ Strongly Agree
◯ Agree
◯ Undecided
◯ Disagree
◯ Strongly Disagree

33 I feel comfortable treating lesbian, gay, bisexual and queer-identified patients

◯ Strongly Agree
◯ Agree
◯ Undecided
◯ Disagree
◯ Strongly Disagree

35 I believe it is the responsibility of all healthcare providers to care for LGBTQ patients.

◯ Strongly Agree
◯ Agree
◯ Undecided
◯ Disagree
◯ Strongly Disagree

36 I can tell if my patient is lesbian, gay, bisexual or queer by looking at them.

◯ Strongly Agree
◯ Agree
◯ Undecided
◯ Disagree
◯ Strongly Disagree

30 My school/program has incorporated LGBTQ related content into a variety of courses.

◯ Strongly Agree
◯ Agree
◯ Undecided
◯ Disagree
◯ Strongly Disagree

37 I can tell if my patient is trans or gender-non-conforming by looking at them.

◯ Strongly Agree
◯ Agree
◯ Undecided
◯ Disagree
◯ Strongly Disagree

38 I feel comfortable discussing sexual health with my patients.

◯ Strongly Agree
◯ Agree
◯ Undecided
◯ Disagree
◯ Strongly Disagree

39 It is more challenging to discuss sexual health with LGBTQ patients than with heterosexual or non-transgender (cis-gender) patients.

◯ Strongly Agree
◯ Agree
◯ Undecided
◯ Disagree
◯ Strongly Disagree

31 My instructors demonstrate competency in caring for LGBTQ patients.

◯ Strongly Agree
◯ Agree
◯ Undecided
◯ Disagree
◯ Strongly Disagree

34 I feel comfortable treating trans-identified patients.

◯ Strongly Agree
◯ Agree
◯ Undecided
◯ Disagree
◯ Strongly Disagree

40 I am interested in receiving further education at Penn about LGBTQ health issues.

◯ Strongly Agree
◯ Agree
◯ Undecided
◯ Disagree
◯ Strongly Disagree
